# Unmanageable Cerebrospinal Fluid Leakage With Eosinophilic Meningitis in a Gliadel Wafer Implant Patient

**DOI:** 10.7759/cureus.59718

**Published:** 2024-05-06

**Authors:** Yuki Kawaguchi, Shunya Hanakita, Shinsuke Yoshida, Tomoko Ikemoto, Soichi Oya

**Affiliations:** 1 Neurosurgery, Saitama Medical Center, Saitama Medical University, Kawagoe, JPN

**Keywords:** drug-induced lymphocyte stimulation test, pseudomeningocele, gliadel, eosinophilic meningitis, carmustine wafer

## Abstract

Gliadel wafer implants (Eisai Inc., Woodcliff Lake, NJ, USA) have shown their efficacy in prolonging survival in patients with malignant gliomas. The safety of Gliadel wafers has also been reported; however, there is a certain risk of adverse events. We present a rare case of refractory cerebrospinal fluid (CSF) leakage with eosinophilic meningitis in a patient with glioblastoma who underwent tumor resection with Gliadel wafer implants. A 60-year-old man presented with a glioblastoma in the right temporal lobe. The patient underwent tumor resection with Gliadel wafer implants. During the postoperative course, the patient presented with intractable CSF leakage and the development of a pseudomeningocele. A delayed rise in blood and CSF eosinophil count (a few weeks after the primary operation) and positive drug-induced lymphocyte stimulation test (DLST) results against the Gliadel wafer led to the diagnosis of an allergic reaction to these implants. Removal of the Gliadel wafers resolved the eosinophilic reaction; however, the patient subsequently required a shunt procedure for persistent hydrocephalus. This case highlights the importance of investigating rare causes of refractory CSF leakage and hydrocephalus due to allergic reactions to Gliadel wafers. Delayed elevations of eosinophils in blood and CSF tests may lead to a diagnosis of eosinophilic meningitis. DLST against Gliadel wafers is also useful for diagnosis when it is available. To control the hydrocephalus, not only the shunt procedure but also wafer removal must be considered; however, patients with limited life expectancy are generally hesitant to undergo such additional procedures.

## Introduction

The most common primary approach and standard treatment strategy for malignant gliomas is maximal safe tumor resection, followed by concomitant chemoradiation with temozolomide (Stupp protocol) [1]. In addition to the Stupp protocol, carmustine wafers (Gliadel wafer, Eisai Inc., Woodcliff Lake, NJ, USA), hereafter referred to as Gliadel, implanted in the tumor resection cavity have shown efficacy as the treatment for malignant glioblastomas [2-4]. In vivo studies have shown that the Gliadel wafer releases carmustine slowly over two to three weeks, mainly during the first five to seven days, and degrades over six to eight weeks [5]. The safety of Gliadel implants in patients with malignant glioma has been widely reported, although some adverse events (AEs) and adverse drug reactions (ADRs) have been noted [3,4,6]. AEs and ADRs with Gliadel implants include various symptoms such as postoperative cerebral edema, pyrexia, seizures, and wound infection [6]. Any symptoms that cause a delay in adjuvant therapy for patients with malignant gliomas should be avoided because the prognosis of these tumors is generally limited [7].

Here, we present a rare case of refractory cerebrospinal fluid (CSF) leakage forming a pseudomeningocele during the postoperative course of primary surgery with a Gliadel wafer implant for glioblastoma. An allergic reaction to the Gliadel wafer was the suspected cause of eosinophilic meningitis, which was confirmed by a drug-induced lymphocyte stimulation test (DLST) performed on the blood and CSF. Wafer removal effectively reduced allergic reactions; however, the patient eventually required a shunt procedure for persistent hydrocephalus.

## Case presentation

**Figure 1:**
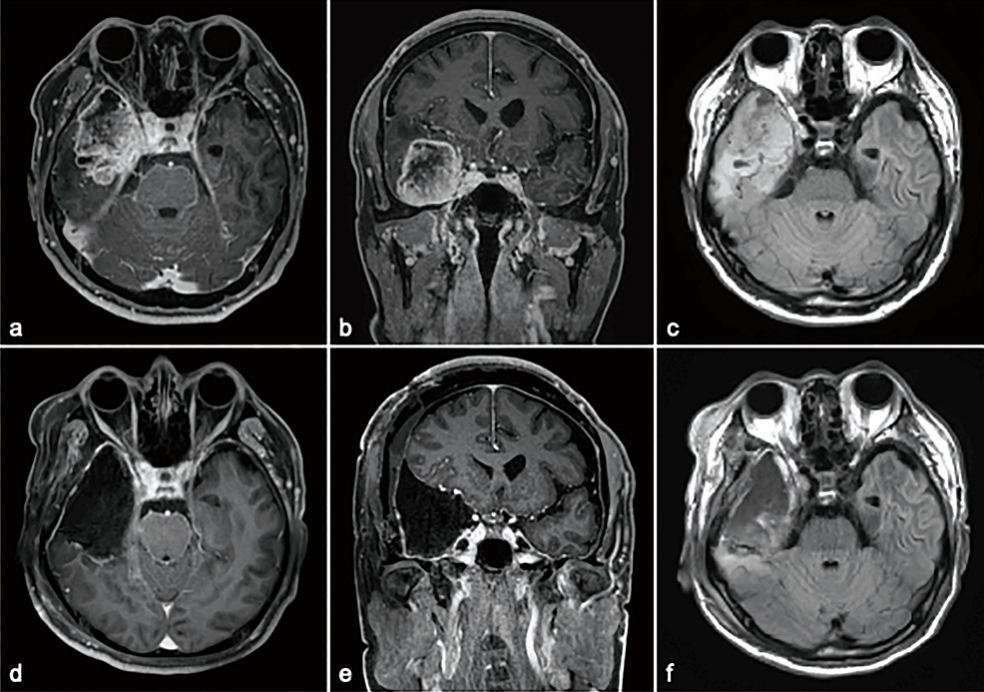
Series of MRI during the pre and postoperative course (a and b) T1-weighted imaging enhanced with gadolinium contrast showed a lesion of 5 cm in diameter with irregular margins and indistinct borders with heterogeneous contrast enhancement on axial and coronal views. (c) Peritumoral edema was identified as a hyper-intense signal on FLAIR imaging. Postoperative MR imaging presented gross total tumor removal (d and e), including the peritumoral high-intensity region on FLAIR imaging (f). FLAIR: fluid-attenuated inversion recovery

A 60-year-old man presented with seizures and a disturbance of consciousness. MR imaging revealed an intra-axial mass with edema in the right temporal lobe (Figure 1).

**Figure 2:**
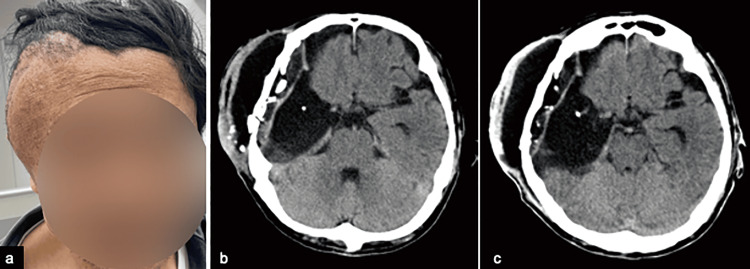
Pseudomeningocele at the surgical site (a)The patient presented pseudomeningocele that gradually worsened two weeks after tumor resection. (b) CT showed intractable subcutaneous effusion that gradually worsened in weeks after the primary surgery. (c) Pseudomeningocele was not resolved despite the placement of lumbar drainage for two weeks.

A T1-weighted image enhanced with gadolinium contrast showed a lesion 5 cm in diameter with irregular margins, indistinct borders, and heterogeneous contrast enhancement (Figure 1). Based on the radiological examination, a malignant glioma was suspected, and the patient underwent tumor resection. Intraoperatively, the ventricle was slightly opened, and ventriculoplasty was performed using cellulose oxide sheets. After confirming gross total resection, eight Gliadel wafers were placed and secured with an absorbable hemostat (oxidized regenerated cellulose) and fibrin glue in the extraction cavity. The dura mater was closed in a standard manner using fibrin glue and polyglycolic acid mesh. Postoperatively, neurological symptoms did not worsen, and contrast-enhanced magnetic resonance imaging performed one day after surgery showed complete removal of the contrast-enhanced lesion (Figure 1). Histopathological examination revealed a glioblastoma. Notably, the patient presented with an intractable subcutaneous effusion that gradually worsened two weeks after primary surgery (Figure 2).

**Figure 3:**
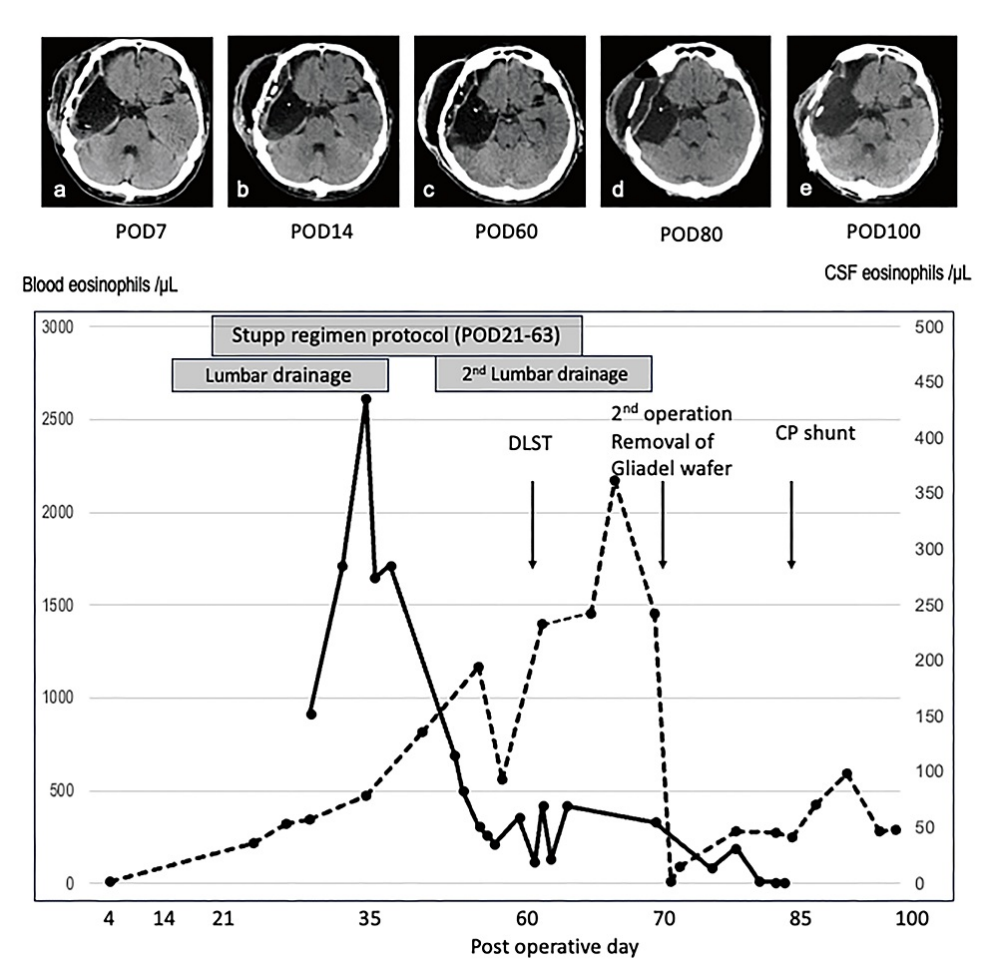
Graph of eosinophil count in the blood and cerebrospinal fluid (CSF) with a series of computed tomography (CT) scans during the postoperative course (a&b) CT showing a pseudomeningocele within one week after the primary surgery, which gradually worsened two weeks after the surgery. (c) Despite treatment with medication and lumbar drainage for several weeks, the CSF leakage did not improve. (d)The Gliadel wafer was removed, but the pseudomeningocele persisted. (e) The shunt procedure was then performed, and the patient did not present a further recurrence of leakage. Graphs of eosinophil counts in blood and CSF tests. An elevation in blood eosinophil count gradually appeared two weeks after the initial surgery. Eosinophil meningitis was suspected based on delayed onset elevation of eosinophils in the CSF test. A drug-induced lymphocyte stimulation test proved that the patient was allergic to the Gliadel wafer, and the Gliadel wafer was removed 10 weeks after the initial surgery. Immediately afterward, the blood eosinophil count decreased markedly, whereas the pseudomeningocele remained after Gliadel removal.

As the increasing subcutaneous effusion appeared to be due to CSF leakage, lumbar drainage was attempted to resolve the CSF leakage two weeks after the initial surgery. At the same time, laboratory examination of the CSF showed an increase in the cell count as follows: cell counts 305 cells/μL (neutrophils 5%, lymphocytes 25%, eosinophils 50%) and sugar 59 mg/dL. The indicated CSF pressure was 11 mmHg. Therefore, we considered the results to be in line with the usual postoperative course, and no further treatment other than lumbar drainage was considered (Figure 3). Although we recognized such subcutaneous formation as an unfavorable condition and there was an increased risk of wound dehiscence during radiotherapy, adjuvant treatment according to the Stupp protocol was started with chemoradiotherapy 21 days after the primary surgery to avoid treatment delay. The treatment course is also summarized in Figure 3.

**Figure 4:**
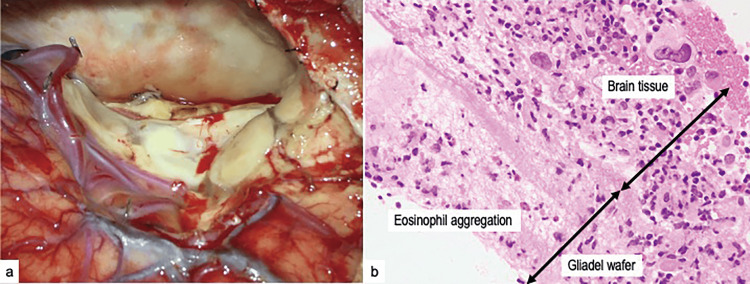
Intraoperative view of Gliadel removal and pathological findings (a) Intraoperative view during removal of the Gliadel wafer. Pulpy-shaped Gliadel wafers strongly adhered to the surface of the tumor cavity. Pathological findings on hematoxylin and eosin staining. (b) Gliadel and necrotic tissues (b). Adjacent to the necrotic tissue, granulation tissue with various inflammatory cells was observed, and histiocytes had infiltrated a large area of eosinophils.

However, the pseudomeningocele did not improve despite lumbar drainage for two weeks. Thirty-five days after the primary resection, blood and CSF tests showed increased eosinophils, both in the white blood cell count (5900 cells/μL, 13.8% eosinophils) and in the CSF (565 cells count/μL, neutrophils 3%, lymphocytes 12%, eosinophils 77%), with a pressure of 35 mmHg. Hence, we suspected an allergic reaction to a certain substance, especially to the Gliadel wafer. We attempted corticosteroid medication (prednisolone 5 mg/day) and repeated lumbar drainage placement; however, these treatments failed. The cause of the allergy was investigated, and a drug-induced lymphocyte stimulation test (DLST) on a Gliadel wafer using CSF and blood yielded positive results. Although the patient developed a massive pseudomeningocele, initial radiochemotherapy was completed as planned. Ten weeks after the initial surgery, the patient underwent reoperation to remove the Gliadel wafers. Intraoperative findings revealed that a part of the dural suture had ruptured, resulting in CSF leakage. The surgical site was divided into several compartments: subcutaneous space, subdural space, and tumor-resected cavity. The previously placed Gliadel wafers adhered strongly to the surface of the extraction cavity but were only removed. The wall of the extraction cavity was aspirated to scrape the entire circumference (Figure 4a). The dura mater was closed by a water-tight dural suture using a pericranium patch and covered with fibrin glue and polyglycolic acid mesh.

**Figure 5:**
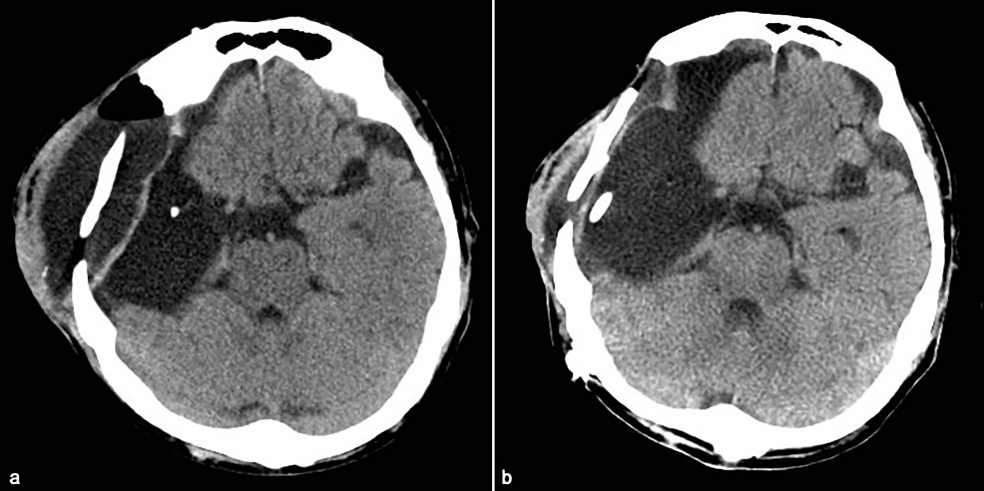
Postoperative CT after removal of Giliadel wafer implants (a) CT showing the CSF leakage remaining for three weeks even after removal of the Gliadel wafer implants. (b) A subdural-peritoneal shunt was performed three months after the primary tumor resection, and the CSF leak was successfully resolved.

Pathological findings revealed the Gliadel and necrotic tissues. Pathological findings included adjacent necrotic tissue, granulation tissue with various inflammatory cells, and infiltration of histiocytes into large areas of eosinophils (Figure 4b). During the postoperative course, the eosinophil ratio in the blood and CSF decreased immediately after the second operation. Contrary to expectations, the CSF leakage persisted for three weeks after the second operation. Finally, a subdural-peritoneal shunt was placed three months after the first procedure, and the CSF leak was successfully resolved (Figure 5). The patient was discharged four months after the first surgery with a Karmofsky Performance Status of 60. During the follow-up period, the patient did not present a recurrence of CSF leakage; however, he died of respiratory failure due to lung metastasis six months after the first surgery.

## Discussion

Gliadel wafers are effective as a local treatment for high-grade gliomas by targeting residual tumors at the resection margins. In a double-blind, randomized phase III study of 240 patients with a first-episode, high-grade glioma, the median overall survival (mOS) was significantly longer in the Gliadel group (13.9 months) than in the placebo group (11.6 months) [8]. In contrast to the benefits of the Gliadel implant, which resulted in a significantly longer median over survival, AEs and ADRs associated with Gliadel wafer placement have been well-reported in the literature [3,4,6]. Most AEs and ADRs included cerebral edema, convulsions, pyrexia, paresis, and wound-healing complications [3,6]. According to a recent literature review, Gliadel wafer removal was rare; only 0.39% of patients in previous studies required removal, and most removals were due to infection at the surgical site [3]. In addition, a recent study from Japan reported the incidence of hydrocephalus as 1.7%, and the cause of hydrocephalus was considered to be ADRs [6].

In the present case, subcutaneous CSF leakage started within one week after the primary resection. The elevation of eosinophils in the CSF was overlooked two weeks after the primary resection and Gliadel implant. In addition, there was a definite time lag before the diagnosis of eosinophilic meningitis owing to the late onset of elevated eosinophil counts in both CSF and blood tests. A previous study reported a similar case of eosinophilic meningitis induced by a Gliadel wafer, which also required wafer removal and a shut procedure to control hydrocephalus [9]. In the previous case, the DSLT was negative for a Gliadel wafer itself but positive for prolifeprosan, which is a biodegradable copolymer used for the gradual release of carmustine from Gliadel wafers. Then, this case is the first to have a positive result of DLST on a Gliadel wafer using CSF and blood. Although the exact cause of allergic reactions related to a Gliadel wafer implant could be different between the previous report and our case, the delay in the elevation of eosinophil count observed 25 days after Gliadel wafer implantation was assumed to reflect gradual degradation over weeks in both cases [9]. Meanwhile, our case showed intractable CSF leakage as a pseudomeningocele that was summed up as an early allergic reaction within the first week, persisting for three months (Figure 3). The allergic reaction induced by Gliadel wafers may lead to eosinophilic meningitis and aggravate dysfunction of CSF circulation, which was originally one of the famous AEs of Gliadel implants that may result in intractable pseudomeningocele formation [6]. Removal of the Gliadel wafer implant and surrounding inflamed brain tissue contributed to a reduction in eosinophilia; however, this was insufficient to control the hydrocephalus, and a shunt procedure was required, as previously reported [9]. Even after the removal of the Gliadel wafer, CSF control may have been challenging for several reasons. Theoretically, carmustine extends up to 5 cm from the drug implant site [10]. Because we generally attempt to achieve maximal tumor resection during the initial surgery, additional resection adjacent to the previous tumor cavity with a margin of 5 cm is challenging because of the risk of new complications. Furthermore, according to the intraoperative findings during the second surgery, the initial surgical site was divided into serval compartments. Therefore, even after the aggressive removal of the Gliadel wafer and surrounding brain tissue, some degrading wafers could remain. Second, the ventricle in the present case was slightly opened, whereas it was immediately closed during primary resection. This may be related to the potential dysfunction of the CSF circulation, resulting in one of the causes of hydrocephalus.

As a local chemotherapy, the Gliadel wafer plays its role within three weeks after implantation and degrades the following three to four weeks [10]. The Stupp protocol should not be interrupted once it has started. However, intractable hydrocephalus with signs of eosinophilic meningitis is so intractable that wafer removal is prioritized over the completion of the treatment protocol when it causes severe complications, especially after mostly finishing its role as a local therapy.

## Conclusions

We present a rare case of irreversible hydrocephalus resulting from inflammation triggered by Gliadel. To lead to the diagnosis of an allergic reaction to a Gliadel wafer, DLST against blood and/or CSF must be useful. Previous reports and the present case insisted on the importance of the shunt procedure in addition to the removal of Gliadel wafers to control CSF circulation. Only the removal of Gliadel wafers must be ineffective to resolve the hydrocephalus. As local chemotherapy, the Gliadel wafer provided control release for approximately three weeks. Thus, wafer removal can be considered after the first three weeks, after which complications that hinder the Stupp protocol occur because a long period of hospitalization is not preferable for patients with limited life expectancy.
